# Severe Cardiorespiratory and Neurologic Symptoms in a Neonate due to Mepivacaine Intoxication

**DOI:** 10.1155/2019/4013564

**Published:** 2019-07-25

**Authors:** Maurike de Groot-van der Mooren, Sabine Quint, Ingmar Knobbe, Doug Cronie, Mirjam van Weissenbruch

**Affiliations:** ^1^Department of Neonatology, Emma Children's Hospital and VU University Medical Center, Boelelaan 1117, Amsterdam 1081 HV, Netherlands; ^2^Department of Pediatric Cardiology, Emma Children's Hospital and VU University Medical Center, Boelelaan 1117, Amsterdam 1081 HV, Netherlands; ^3^Department of Obstetrics and Gynaecology, Onze Lieve Vrouwe Gasthuis, Jan Tooropstraat 164, Amsterdam 1061 AE, Netherlands

## Abstract

Local anesthesia with mepivacaine is used for vaginal deliveries and for minor surgeries of the vagina and perineum as repair of an episiotomy or perineal laceration. Neonatal intoxication caused by local anesthesia with mepivacaine for maternal episiotomy has been rarely reported. We present a case of a term female infant with unexplained cardiorespiratory distress and several neurologic findings, including seizures, one hour after birth. Electrocardiogram showed a second-degree atrioventricular block and a left-bundle branch block. Blood measures in the patient revealed a high mepivacaine level following local anesthesia for maternal episiotomy. Because of the increasing practice of local anesthesia, high awareness for neonatal intoxication and further research in safe elimination therapy in neonates is needed.

## 1. Introduction

Local anesthetics with mepivacaine are used for vaginal deliveries and for minor surgeries of the vagina and perineum as repair of an episiotomy or perineal laceration [1]. Neonatal complications are uncommon. We here present a newborn with sudden respiratory, neurologic, and cardiac signs, shortly after birth, following maternal episiotomy after perineal nerve block with mepivacaine 100 milligrams (mg) per milliliter (ml) (10 mg/ml, 10 ml) during labor.

## 2. Case Presentation

A term female infant who was born in a referral hospital was transferred to our neonatal intensive care unit (NICU) after unexpected signs of hypothermia (35. 3°C) and an episode of recurrent apnea, irregular respiration, bradycardia, signs of hypotonia, and suspected convulsions one hour after birth.

In the mother, a healthy 27-year-old primigravida, labor was induced at 38 weeks and 3 days, because of suspicion of intrauterine growth restriction. During 34 weeks, pregnancy abdominal circumference (AC) was at the 37th percentile (p37) and estimated fetal weight (EFW) at the p18. At 35 weeks, pregnancy AC was at the p17 and EFW at the p11. Besides the fetal growth restriction, no other abnormal findings were found during pregnancy. The mother had a remarkable short stature, 149 centimeters (cm), and weighed less than 45 kilograms (kg) but was healthy. Family anamnesis revealed no neurological or cardiac diseases. There was no history of maternal medication use or drugs abuse during pregnancy. There were no risk factors for infection during pregnancy and birth.

The mother underwent episiotomy after perineal nerve block with 100 mg of mepivacaine (10 ml, 10 mg/ml), about twenty minutes before delivery. External intrapartum fetal monitoring showed no signs of fetal distress. The time interval between delivery and cord clamping was five minutes. Umbilical-cord blood gas analysis was normal.

A girl was born with Apgar scores 9 and 10 after 1 and 5 minutes, respectively. She had a birthweight of 2854 grams (p20), a birth length of 50 cm (p67), and a head circumference of 33 cm (p23), all in normal range. Breastfeeding was initiated immediately. Because of the clinical deterioration one hour after birth, the newborn was first admitted to the neonatology ward of the referral hospital. During the episodes of apneas, fasciculation of the tongue, tremor of the lips, and smacking movements of the mouth were noticed. Extremities were put in flexion, with pointed toes, hands in fists, and pupillary mydriasis fixed to light. Before the apnea, a short crying was observed. In between these seizures, she was hypotonic but normal neonatal reflexes could be provoked. Venous access was difficult to obtain, so an umbilical venous catheter was inserted and phenobarbital 20 mg/kg was given intravenously. One and a half hours after birth, there was a sudden decrease in the heart rate of 80–90 beats/minute. Atropine was given but had no effect on the heart rate. The blood pressure and body temperature were stable and in normal range.

Based on the above findings, she was intubated and thereafter transferred to our NICU. Physical examination showed an encephalopathic, nonresponsive neonate on the ventilator, with bradycardia of 65 beats/minute and well saturated. Neurological examination showed a decorticated posture, hypotonia and pupillary mydriasis fixed to light, without brainstem reflexes.

Laboratory tests showed normal glucose, blood cell count, electrolytes, and kidney function. In addition, bilirubin 64 micromol per liter (*μ*mol/L), creatinine kinase 854 units per liter (U/L), and lactate 1.5 millimol per liter (mmol/L) were normal. ALT was raised (435 U/L), AST and gamma GT were hemolytic, and ammoniac was slightly elevated to 120 *μ*mol/L, however, normalized to 64 *μ*mol/L 5 hours after birth. A low level of C-reactive protein was found (3 milligrams per liter (mg/L)).

Brain monitoring using 2-channel amplitude-integrated EEG (aEEG) showed a status epilepticus and again she was treated with phenobarbital 10 mg/kg intravenously. The bradycardia recovered after administering phenobarbital suggesting the bradycardia was related to convulsions.

**Figure 1:**
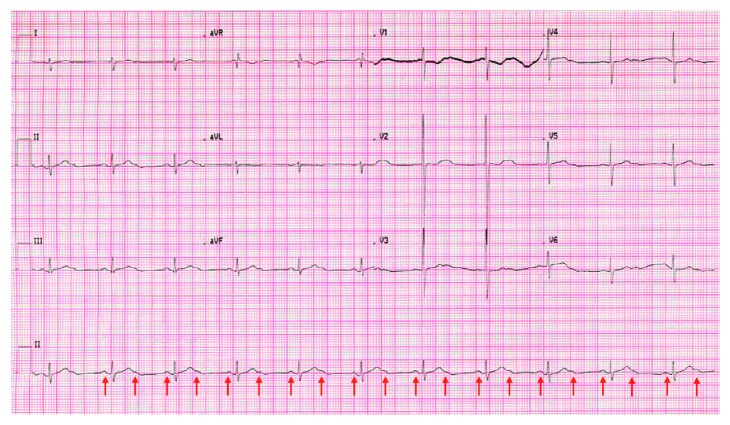
ECG during bradycardia showed a second-degree AV block with an atrial frequency of 130/min and a ventricular frequency of 65/min. QRS 76 ms; QTc 590 ms. Red arrows are pointed towards the *P*-waves, at times hidden in the *T*-waves. Settings 25 mm/s; 10 mm/mV.

Some hours later and during local transportation to the radiology department to undergo a magnetic resonance imaging (MRI) of the brain, again a bradycardia was present. aEEG at that time could not be registered. Because bradycardia was thought to be a symptom of a convulsion, a third phenobarbital dose was administered intravenously (10 mg/kg). However, it did not show any effect on the heart rhythm. Evaluation of the electrocardiogram (ECG) showed a second-degree atrioventricular (AV) block (Figure 1).

**Figure 2:**
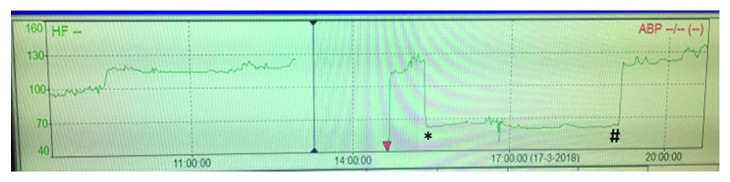
Illustration of the curve of the heartbeat with episodes of a sudden bradycardia (^*∗*^) and spontaneous recovery to normal AV conduction (#). *Y*-axis: heart rate in beats per minute; *X*-axis: time in hours.

**Figure 3:**
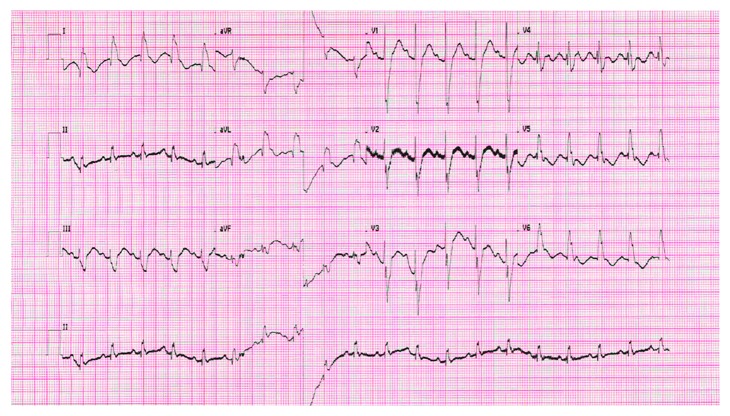
ECG on the right showed normal heart frequency (131 beats/minute) with 1 : 1 AV conduction, with a left bundle branch block (QRS 120 ms). Settings 25 mm/s; 10 mm/mV.

Figure 2 shows the heart rate over time with a sudden decline in the heart rate and sudden recovery from 2 : 1 AV conduction to normal AV conduction. A 21-channel video EEG at that time showed no epileptic activity, suggesting bradycardia was not related to convulsions. A few hours later, bradycardia resolved spontaneously and ECG showed a left bundle branch block (Figure 3).

Empirical antibiotics were started, but cultures of blood and cerebrospinal fluid remained negative. Antibiotics were stopped after 48 hours. Also, liquor polymerase chain reaction (PCR) on neurotropic viruses was negative. Metabolic screening in cerebrospinal fluid and urine was normal. Brain ultrasound and MRI/magnetic resonance spectroscopy (MRS) at days 1 and 2 showed no abnormalities. The infant improved further, and on the second day of life, her physical and neurologic examination restored to normal whereafter she was extubated. The ECG normalized. Seven days later, the neonate was fully recovered and could be discharged from the NICU. In retrospection, an intoxication with a local anesthetic was suspected. The consulted obstetrician confirmed the use of mepivacaine for a perineal infiltration during delivery. Therefore, blood samples obtained from the first days of life were analyzed. On the first day, the mepivacaine level was 20 mg/L, and on the second day, it was 2 mg/L. There was no injection site visible on the scalp. The mother did not show any signs of intoxication during or after delivery. Following up at 8 months of age, the infant's neurodevelopmental examination was in normal range.

## 3. Discussion

Apnea, bradycardia, prolonged QRS complexes, cyanosis, hypotonia, pupillary mydriasis fixed to light, and seizures are characteristic features for an intoxication with local anesthetics in neonates. The mother in this case received local anesthesia with mepivacaine for maternal episiotomy. The neonate had typical symptoms of an intoxication, and the blood sample on the first day revealed a mepivacaine level of 20 mg/L. Toxic effects in neonates have been described with levels as low as 3 mg/L. Furthermore, the neonate was fully recovered on the second day, which was coherent with a mepivacaine level of 2 mg/L.

Perinatal infection or metabolic causes were excluded. Although perinatal asphyxia may also explain these symptoms, differences in the early course of patients can differentiate for an intoxication. Hypoxic-ischemic encephalopathy caused by perinatal asphyxia is usually not characterized by high Apgar scores followed by a symptomless period after birth before the onset of hypotonia, tonic seizures, and arrest of spontaneous respiration or with fixed dilated pupils and eyes fixed in the oculocephalic reflex. Furthermore, brain imaging and blood results were not suggestive for asphyxia during pregnancy or labor. The onset of all the signs observed in our case might therefore be caused by intoxication from local anesthesia [2].

Mepivacaine similar to lidocaine is an amide-type local anesthetic agent. It reversibly blocks the sodium channels of nerve fibers, thereby inhibiting the conduction of nerve impulses. As mepivacaine affects the function of skeletal muscles, it reduces conduction of the AV node (second-degree AV block) and conduction over the His bundle causing a left bundle branch block. Without the knowledge of mepivacaine treatment in our case, a second-degree AV block and pseudoblock with QTc around 590 ms were considered. However, in mepivacaine intoxication, AV conduction disturbances are much more likely than prolonged repolarization (pseudoblock). Mepivacaine is known to cross the placental barrier and can affect the fetus. Two possibilities for transmission are described in the literature: first, trans-placental transmission following perineal infiltration for maternal episotomy [3] and second, accidental injection (in the fetal scalp) during infiltration for paracervical and pudendal blocks during labor [4].

Lidocaine intoxication is better known in neonatal intoxication after local anesthesia [5, 6]. Recently, a case report described a toxic lidocaine level in a cord blood sample, so trans-placental intoxication of lidocaine was verified [7]. In our case, the mepivacaine level was not examined in the cord blood; however, trans-placental intoxication seems to be most likely. The perineal infiltration technique was applied lege artis, keeping the fingers between the needle and the fetal skull. Throughout the procedure, the perineum bulged as the mepivacaine was injected. Because no skull laceration was seen in our case, direct injection in the skull seems highly unlikely but cannot completely ruled out because of a hairy skull. However, the birth attendant confirmed this did not happen.

Pharmacokinetic studies show diverse fetal/maternal lidocaine ratios depending on the route of administration (perineal infiltration, lumbar epidural anesthesia, pudendus block, or paracervical block). Because mepivacaine is like lidocaine, an amide-type local anesthetic agent, the same explanations may be given for the high fetal/maternal ratio after perineal infiltration of mepivacaine as for lidocaine. First, the local anesthesia is injected in a highly vascularized zone. Second, there is anatomic proximity of the perineum to the fetus. Third, mepivacaine is a weak base which will rapidly cross the placenta in the unionized state. Because of fetal acidosis in the second stage of labor, there is an increased conversion to ionized mepivacaine in the fetus; the ratio ionized to unionized mepivacaine rises. This leads to an accumulation of mepivacaine in the fetus. This phenomenon is called “trapping” of the anesthesia in the ionized form. This explains the strong correlation between the fetal/maternal ratio and the length of the second stage of labor and in fetal asphyxia. Additionally, half-life time of mepivacaine is at least three times longer in neonates than in adults, respectively, 9 hours versus 2 to 3 hours. Toxic effects in neonates have been described with levels as low as 3 mg/L [8]. Because local anesthetics accumulate preferentially in acidic media, elimination can be enhanced with gastric lavage (however, the risk for aspiration should be taken into account) and forced diuresis. Elimination by exchange transfusion is possible too. Furthermore, intravenous intralipid administration is described to resolve toxicity in neonates caused by local anesthetic procedures. Intralipid is an emulsion of soybean oil in water, predominantly neutral triglycerides, made isotonic with glycerin. In blood, these fat droplets form a lipid compartment, separate from the plasma aqueous phase, into which a lipophilic substance like mepivacaine might dissolve (lipid sink therapy). A concentration gradient develops between tissue and blood which causes local anesthetics to move from the heart or brain (areas of high concentrations) to the “lipid sink”. Because of the increasing practice of local anesthesia, further research in safe elimination therapy as intralipid in neonates is therefore needed [9]. By the time the diagnosis was established in this current case, the patient was already recovered and elimination therapy was not necessary.

Exact morbidity and mortality of infants following intoxication by local anesthetics is not known. Long-term follow-up studies documenting neurologic and developmental outcome are necessary; however, this characteristic syndrome after maternal local anesthetics is rare and may therefore often be unrecognized.

This case presents a term newborn with respiratory, cardiac, and neurologic signs shortly after birth most probably caused by intoxication with mepivacaine following perineal infiltration for maternal episiotomy. Although this is an uncommon complication, we advise that in case of unexplained symptoms, intoxication involving the fetus during labor has always to be taken into account.
